# Endoscopic ultrasound‐guided choledochoduodenostomy without fistula dilation using a stent with a 5.9‐Fr delivery system: Comparison to a conventional procedure with fistula dilation

**DOI:** 10.1002/deo2.56

**Published:** 2021-09-29

**Authors:** Takehiko Koga, Susumu Hijioka, Yosikuni Nagashio, Akihiro Ohba, Yuta Maruki, Motohiro Yoshinari, Yuya Hisada, Shota Harai, Hidetoshi Kitamura, Kosuke Maehara, Yumi Murashima, Yuki Kawasaki, Shun Kawahara, Kotaro Takeshita, Natsumi Yamada, Tomoyuki Satake, Shunsuke Kondo, Chigusa Morizane, Hideki Ueno, Takuji Okusaka, Yutaka Saito

**Affiliations:** ^1^ Department of Hepatobiliary and Pancreatic Oncology National Cancer Center Hospital Tokyo Japan; ^2^ Endoscopy Division National Cancer Center Hospital Tokyo Japan

**Keywords:** endoscopic ultrasound‐guided biliary drainage, endoscopic ultrasound‐guided choledochoduodenostomy, EUS, EUS‐BD, EUS‐CDS

## Abstract

**Objectives:**

To evaluate the feasibility and safety of endoscopic ultrasound‐guided choledochoduodenostomy (EUS‐CDS) without fistula dilation using a novel self‐expandable metal stent (SEMS).

**Methods:**

This retrospective study examined patients who underwent EUS‐CDS for malignant distal biliary obstruction between October 2017 and May 2021 at the National Cancer Center, Japan. The primary outcome was a technical success without fistula dilation. Secondary outcomes were the overall technical success, clinical success, adverse events (AEs), procedure time, recurrent biliary obstruction (RBO), and time to RBO (TRBO).

**Results:**

Forty‐one patients were enrolled; 31 patients underwent EUS‐CDS with fistula dilation using a conventional SEMS with 7.5–8.5‐Fr delivery system (conventional SEMS group), and 10 patients underwent EUS‐CDS without fistula dilation using the novel SEMS with a 5.9‐Fr delivery system (novel SEMS group). In the novel SEMS group, the rate of technical success without fistula dilation was 90%. There were no differences in overall technical success (100% vs. 97%, *p* = 1.00), clinical success (80% vs. 90%, *p* = 0.58), and overall AEs (10% vs. 23%, *p* = 0.65) rates between the novel and conventional SEMS groups. In the novel SEMS group, no early AEs were observed and no bile leakage into the abdominal cavity was observed on the computed tomography scan after the procedure. The median procedure time was significantly shorter in the novel SEMS group (17 min vs. 24 min, *p* = 0.03). RBO and median TRBO did not differ between the 2 groups.

**Conclusions:**

EUS‐CDS without fistula dilation using the novel SEMS with a 5.9‐Fr delivery system is technically feasible, straightforward, quick, and safe.

## INTRODUCTION

Endoscopic retrograde cholangiopancreatography (ERCP) is widely performed as a drainage procedure for patients with malignant biliary obstructions. Recently, endoscopic ultrasound‐guided choledochoduodenostomy (EUS‐CDS) has also been used as primary drainage for unresectable malignant distal biliary obstructions.^1^, ^2^, ^3^, ^4^, ^5^, ^6^, ^7^, ^8^ The advantages of EUS‐CDS are highly technical and clinical success rates and the absence of the risk of pancreatitis. However, the rate of adverse events (AEs) is high. The most common AEs are bile leakage and peritonitis, and these are associated with the fistula dilation process in the EUS‐CDS procedure.^9^, ^10^, ^11^ The conventional stents used for EUS‐CDS are partially or fully covered self‐expandable metal stents (SEMSs) with a 7.5–8.5‐Fr delivery system, that requires fistula dilation.^12^ We previously reported a case in which EUS‐CDS without fistula dilation was successfully performed using a novel fully covered SEMS with a 5.9‐Fr delivery system.^13^ Other than this case, there are no other reports on EUS‐CDS using this SEMS. Furthermore, the technical feasibility and safety of EUS‐CDS without fistula dilation are also unclear. Herein, we present the outcomes of the patients who underwent EUS‐CDS without fistula dilation using a fully covered SEMS with a 5.9‐Fr delivery system and compared these outcomes with those of patients who underwent EUS‐CDS using a conventional SEMS with fistula dilation.

## PATIENTS AND METHODS

This retrospective study examined 41 patients who underwent EUS‐CDS for malignant distal biliary obstruction between October 2017 and May 2021 at the National Cancer Center, Japan. EUS‐CDS performed before April 2020 used a conventional fully covered SEMS with a 7.5–8.5‐Fr delivery system, and that after May 2020 used a novel fully covered SEMS with a 5.9‐Fr delivery system. All patients provided informed consent. The study was approved by the Institutional Review Board of the National Cancer Center Hospital, Japan (2018‐1‐149), and was performed in accordance with the principles of the Declaration of Helsinki.

### The conventional SEMS

The stents we used between October 2017 and April 2020 were conventional fully covered SEMSs with 7.5–8.5‐Fr delivery systems (X‐suit NIR; Olympus Medical Systems, Tokyo, Japan, HANAROSTENT; M.I.Tech, Seoul, Korea, and Niti‐S; Taewoong Medical, Seoul, Korea). The choice of the SEMS was per the echoendoscopist's discretion.

### The novel SEMS

**FIGURE 1:**
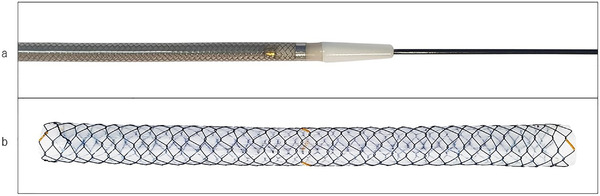
Fully covered self‐expandable metal stent with a 5.9‐Fr delivery system. (a) The outer sheath of the delivery catheter is sized 5.9 Fr with the tapered tip. (b) The expanded stent with a braiding design

The stent we used between May 2020 and May 2021 was a novel type of braided SEMS (HANAROSTENT Benefit; M.I.Tech) (Figure 1). The SEMS was of the fully covered type with an ultra‐thin diameter (5.9‐Fr) delivery system. Compared with conventional HANAROSTENT with an 8.5‐Fr delivery system, which is the same braided fully‐covered SEMS, the stent wire and silicone cover was thinner, and the stent cell size was wider. Therefore, the radial force was down by approximately 40% compared to the conventional HANAROSTENT. The shortening rate after deployment was 30%–40%. The SEMS was available in diameters of 6 and 8 mm and lengths of 6, 8, 10, and 12 cm. During EUS‐CDS, the SEMS of 8 mm in diameter and 6 cm in length was our first choice, because it was considered to have a low risk of migration and good fitness to the bile duct. However, SEMSs of other sizes were also selected based on each individual patient's anatomical condition.

### Procedural technique

**FIGURE 2:**
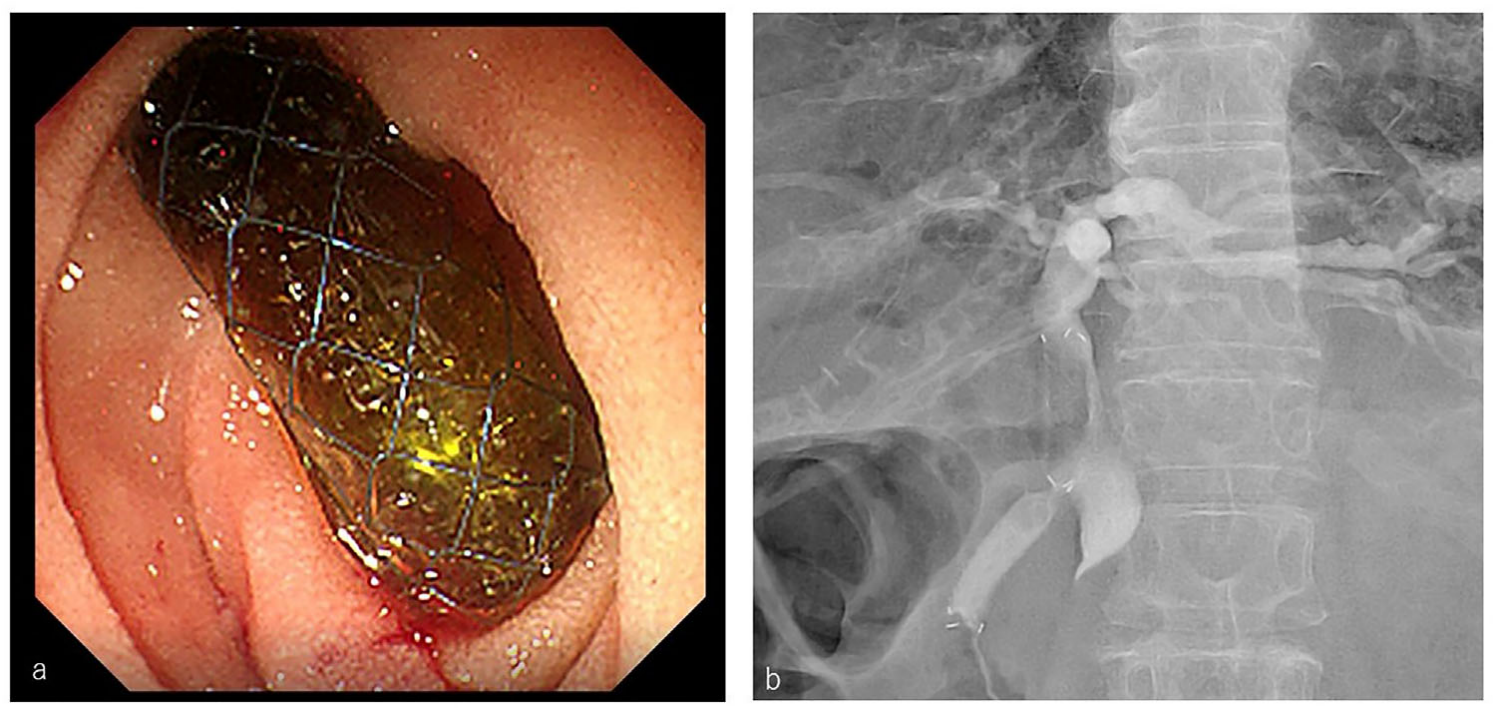
Imaging findings following endoscopic ultrasound‐guided choledochoduodenostomy without fistula dilation. (a) Endoscopic image. (b) Fluoroscopic image

EUS‐CDS was performed by one of the two experts (experience of>50 EUS‐guided biliary drainages) or 12 trainee echoendoscopists (experience of>200 ERCP and >50 EUS‐guided fine‐needle aspirations) under expert supervision. An oblique‐viewing echoendoscope (GF‐UCT240, GF‐UCT260; Olympus Medical Systems, Tokyo, Japan and EG‐580UT; Fujifilm, Tokyo, Japan) or a forward‐viewing echoendoscope (TGF‐UC260J; Olympus Medical Systems) was used to perform EUS‐CDS. The echoendoscope choice was left to the discretion of the echoendoscopist. However, for the cases in which endoscopic ultrasonography screening was performed before the procedure, the angle of the needle insertion, the distance from the puncture point to the hilum, and the scope position were estimated, and the echoendoscope that had greater accessibility with regards these factors were selected. A 19‐gauge needle for EUS‐guided fine‐needle aspiration (EZ shot 3 plus; Olympus Medical Systems and Beacon FNA Exchange System; COVIDIEN, Tokyo, Japan) was used to puncture the extrahepatic bile duct. Subsequently, bile was aspirated via the needle until the diameter of the bile duct was reduced to approximately half, and the hilum was verified using cholangiography. Then, a 0.025‐inch guidewire (VisiGlide2; Olympus Medical Systems and M‐Though; ASAHI INTECC Corp., Tokyo, Japan) was placed in the bile duct. In the patients with the conventional SEMS, the SEMS was inserted after performing fistula dilation using an electrocautery dilator (Cysto‐Gastro Set; Endoflex, Voerde, Germany and Fine025; Medico's Hirata Inc., Osaka, Japan) or a balloon catheter (REN; Kaneka Medical, Osaka, Japan); in the patients with the novel SEMS, the SEMS was directly inserted without a prior fistula dilation process. The SEMS was deployed through the fistula between the extrahepatic bile duct and the duodenal bulb. Finally, the distal end of the SEMS was pushed by the scope and directed toward the anal side to prevent early stent dysfunction^14^ (Figure 2). Abdominal computed tomography (CT) was performed immediately after the procedure to confirm the position of the SEMS, presence of fluid collection, or leakage of the contrast medium (i.e., bile leakage) into the abdominal cavity.

### Definition of outcomes

The primary outcome was a technical success without fistula dilation. The secondary outcomes were: (1) overall technical success, (2) clinical success, (3) AEs, (4) procedure time, (5) recurrent biliary obstruction (RBO), and (6) time to RBO (TRBO). Technical success without fistula dilation was defined as when the novel SEMS was inserted directly without a fistula dilation process and successfully placed. Overall technical success was defined as a successful novel or conventional SEMS placement with or without the fistula dilation process. Procedure time was measured from when the bile duct was punctured to the deployment and positioning of the SEMS. The following definitions were used in accordance with the Tokyo criteria 2014.^15^ Clinical success was defined as a 50% reduction or normalization of total bilirubin level within 14 days after the procedure. AEs were classified as early (occurring within 30 days after the procedure) and late (occurring after 31 days or later). RBO was defined as the occlusion or migration of the SEMS. TRBO was defined as the time between the initial stenting and the occurrence of RBO.

### Statistical analysis

The primary outcome, technical success without fistula dilation, was examined only in the patients using the novel SEMS. The secondary outcome was examined in all patients and compared between the novel and conventional SEMS groups. Continuous variables were expressed using medians and ranges, while categorical variables were expressed as proportions. Continuous variables were analyzed using the Mann‐Whitney *U*‐test. Categorical variables were analyzed using the chi‐square test or Fisher's exact test. The TRBO was estimated using the Kaplan–Meier method and the log‐rank test was used to determine statistically significant differences. A *p*‐value of <0.05 was considered statistically significant. All analyses were performed using SPSS version 25.0 (SPSS Inc.; IBM Corp, Armonk, NY, USA).

## RESULTS

**FIGURE 3:**
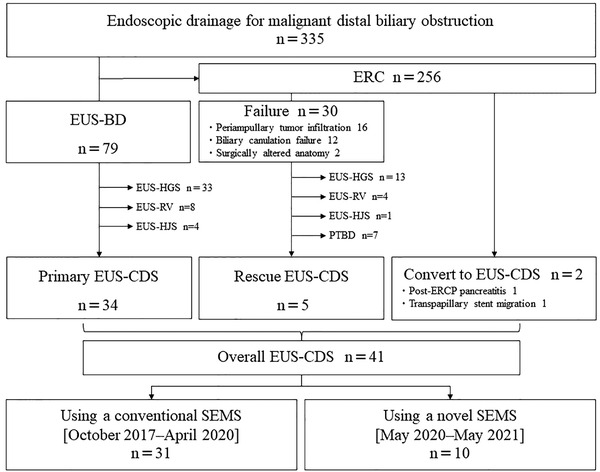
Study flow chart of patients undergoing endoscopic ultrasound‐guided choledochoduodenostomy. ERC, endoscopic retrograde cholangiography; EUS‐BD, endoscopic ultrasound‐guided biliary drainage; EUS‐HGS, endoscopic ultrasound‐guided hepaticogastrostomy; EUS‐RV, endoscopic ultrasound‐guided rendezvous technique; EUS‐HJS, endoscopic ultrasound‐guided hepaticojejunostomy; PTBD, percutaneous transhepatic biliary drainage; EUS‐CDS, endoscopic ultrasound‐guided choledochoduodenostomy; ERCP, endoscopic retrograde cholangiopancreatography

From October 2017 to May 2021, a total of 335 patients with malignant distal biliary obstruction underwent endoscopic biliary drainage. For 34 patients (10%), EUS‐CDS as primary drainage was performed. EUS‐CDS as rescue drainage was performed in five of 30 patients with ERCP failure. Two patients were converted to EUS‐CDS after transpapillary stenting. A total of 41 patients (12%) underwent EUS‐CDS. Of these 31 patients underwent EUS‐CDS using the conventional SEMS (conventional SEMS group) [before April 2020], and 10 patients underwent EUS‐CDS using the novel SEMS (novel SEMS group) [after May 2020] (Figure 3).

### Patients’ characteristics

Table 1 shows the patients’ characteristics. The median age was 68 years (range, 40–82 years). Pancreatic cancer was the most common cause of biliary obstruction (88%).[Table deo256-tbl-0002]

**TABLE 1:** Patients’ characteristics

	Novel SEMS with a 5.9‐Fr delivery system, *n* = 10	Conventional SEMS with 7.5–8.5‐Fr delivery system, *n* = 31	*p*
Median age, years (range)	69 (55–78)	67 (40–82)	0.68
Male, no. (%)	5 (50)	19 (61)	0.71
Cause of distal biliary obstruction, no. (%)			0.60
Pancreatic cancer	10 (100)	26 (84)	
Ampullary cancer	0 (0)	1 (3)	
Metastatic pancreatic tumor	0 (0)	3 (10)	
Metastatic lymph nodes	0 (0)	1 (3)	
Indications for EUS‐CDS, no. (%)			0.26
Primary drainage	10 (100)	24 (77)	
Rescue drainage from ERC failure	0 (0)	5 (16)	
Conversion from transpapillary stenting	0 (0)	2 (6)	
Treatment for the primary tumor, no. (%)			0.83
Chemotherapy	7 (70)	29 (94)	
Best supportive care	3 (30)	2 (6)	
Post‐radiation therapy for primary tumor, no. (%)	1 (10)	1 (3)	0.43
Abdominal CT findings			
Tumor size, median, mm (range)	36.0 (20–69)	30.0 (10–50)	0.20
Ascites, no. (%)	3 (30)	9 (29)	1.00
Duodenal invasion, no. (%)	6 (60)	16 (52)	0.73
Duodenal stent placement, no. (%)	2 (20)	1 (3)	0.14

Abbreviations: CT, computed tomography; ERCP, endoscopic retrograde cholangiopancreatography; EUS‐CDS, endoscopic ultrasound‐guided choledochoduodenostomy; SEMS, self‐expandable metal stent.

**TABLE 2 deo256-tbl-0002:** Details of the endoscopic ultrasound‐guided choledochoduodenostomy (EUS‐CDS) procedure

	Novel SEMS with a 5.9‐Fr delivery system, *n* = 10	Conventional SEMS with 7.5–8.5‐Fr delivery system, *n* = 31	*p*
Scope, no. (%)			0.27
Oblique‐viewing echoendoscope	6 (60)	11 (35)	
Forward‐viewing echoendoscope	4 (40)	20 (65)	
Needle, no. (%)			0.24
19‐gauge EZ shot 3 plus	9 (90)	31 (100)	
19‐gauge Beacon FNA Exchange System	1 (10)	0 (0)	
Guidewire, no. (%)			0.003
0.025‐inch M‐Though	10 (100)	15 (48)	
0.025‐inch VisiGlide2	0 (0)	16 (52)	
Stent diameter × length, no. (%)			0.06
6 mm × 6 cm	1 (10)	2 (6)	
8 mm × 6 cm	8 (80)	13 (42)	
8 mm × 8 cm	1 (10)	1 (3)	
10 mm × 6 cm	0 (0)	14 (45)	
EUS findings			
Diameter of the punctured bile duct, median, mm (range)	11.5 (7–19)	12.0 (8–28)	0.26
Length of the puncture route, median, mm (range)	10.5 (5–14)	6.0 (3–20)	0.01
The first endoscopist, no. (%)			0.04
Trainee	10 (100)	20 (65)	
Expert	0 (0)	11 (35)	

Abbreviations: EUS, endoscopic ultrasonography; EUS‐CDS, endoscopic ultrasound‐guided choledochoduodenostomy; SEMS, self‐expandable metal stent.

All of the 10 patients (100%) in the novel SEMS group and 26 of the 31 patients (84%) in the conventional SEMS group underwent EUS‐CDS as primary drainage. One patient each in both groups had previously received radiation therapy for pancreatic head cancer. Overall, there was no statistically significant difference between the two groups in terms of clinical characteristics.

### Procedural details

The forward‐viewing echoendoscope was used in four of the 10 patients (40%) in the novel SEMS group and in 20 of the 31 patients (65%) in the conventional SEMS group. In the novel SEMS group, the SEMS with 8‐mm diameter and 6‐cm length was commonly used (80%, *n* = 8). In the conventional SEMS group, X‐suit NIR was used in 18 patients (58%), HANAROSTENT in 10 patients (32%), and Niti‐S in two patients (6%). The SEMS with 10‐mm diameter and 6‐cm length was placed in 14 of the 31 patients (45%), and the SEMS with 8‐mm diameter and 6‐cm length was placed in 13 patients (42%). The EUS findings showed that the median diameter of the punctured bile duct was 11.5 mm in the novel SEMS group and 12.0 mm in the conventional SEMS group. The median length of the puncture route (distance from the scope to the puncture point of the bile duct on the EUS images) was 10.5 mm in the novel SEMS group and 6.0 mm in the conventional SEMS group with a significant difference (*p* = 0.01).

### Outcomes

**FIGURE 4:**
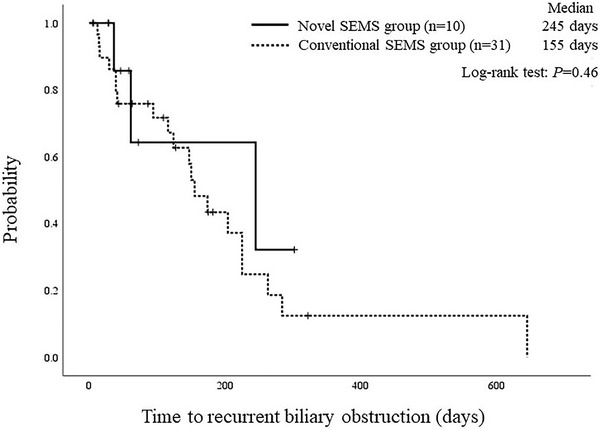
Kaplan–Meier curves with log‐rank test for time to recurrent biliary obstruction in the novel and conventional self‐expandable metal stent (SEMS) groups

Table 3 shows the outcomes of EUS‐CDS. In the novel SEMS group, the rate of technical success without fistula dilation was 90% (9/10). In one patient who required fistula dilation, a forward‐viewing echoendoscope was used. The diameter of the punctured bile duct was 19.0 mm and the length of the puncture route was 10.0 mm. Consequently, the technical success was achieved after additional fistula dilation using a 7‐Fr electrocautery dilator. The overall technical success rate was 100% (10/10) in the novel SEMS group and 97% (30/31) in the conventional SEMS group. The clinical success rate was 80% (8/10) in the novel SEMS group and 90% (28/31) in the conventional SEMS group. The two clinically unsuccessful patients in the novel SEMS group died of multiple organ failure due to the rapid progression of the primary disease. In the novel SEMS group, the overall AE rate was 10% (1/10). One late AE of non‐occlusion cholangitis occurred on postoperative day 46, but there were no cases of early AEs. In the conventional SEMS group, however, early AEs were observed in six (19%) patients, cholecystitis in two, bile peritonitis in one, non‐occlusion cholangitis in one, bleeding in one, and portal vein‐bile duct fistula in one. Furthermore, abdominal CT after the procedure showed that there was no leakage of the contrast medium into the abdominal cavity in the novel SEMS group, whereas 13 (42%) patients in the conventional SEMS group had leakage, showing a significant difference (*p* = 0.02). The median procedure time was significantly shorter in the novel SEMS group than in the conventional SEMS group (17.0 min vs. 24.0 min, *p* = 0.03). Among the novel SEMS group, RBOs were observed in three (30%) patients, distal migration in two (postoperative days 36 and 61), and occlusion in one (day 245). Two patients with migration were treated by placing a new SEMS through the fistula. In the patient with the occlusion, because the technical approach to the fistula was difficult due to progressive duodenal stenosis caused by tumor invasion, EUS‐guided hepaticogastrostomy, and duodenal stent placement were performed. There was no significant difference in the median TRBO between the novel and conventional SEMS group (245 days vs. 155 days, *p* = 0.46) (Figure 4).

**TABLE 3:** Outcomes of endoscopic ultrasound‐guided choledochoduodenostomy (EUS‐CDS)

	Novel SEMS with a 5.9‐Fr delivery system, *n* = 10	Conventional SEMS with 7.5–8.5‐Fr delivery system, *n* = 31	*p*
Technical success without fistula dilation, no. (%)	9 (90)		
Overall technical success, no. (%)	10 (100)	30 (97)	1.00
Clinical success, no. (%)	8 (80)	28 (90)	0.58
Adverse events, no. (%)	1 (10)	7 (23)	0.65
Early adverse events, no. (%)	0 (0)	6 (19)	0.31
Cholecystitis	0	2	
Bile peritonitis	0	1	
Non‐occlusion cholangitis	0	1	
Bleeding	0	1	
Portal vein‐bile duct fistula	0	1	
Late adverse events, no. (%)	1 (10)	1 (3)	0.43
Non‐occlusion cholangitis	1	0	
Bleeding	0	1	
Contrast medium leakage into the abdominal cavity after the procedure	0 (0)	13 (42)	0.02
Procedure time in minutes, median (range)	17.0 (11–25)	24.0 (11–65)	0.03
RBO, no. (%)	3 (30)	20 (65)	0.07
Migration	2 (20)	14 (45)	
Occlusion	1 (10)	6 (19)	
TRBO in days, median (95% CI)	245 (0–526)	155 (117–193)	0.46
Follow‐up period in days, median (range)	52 (5–302)	120 (6–645)	0.17

Abbreviations: CI, confidence interval; EUS‐CDS, endoscopic ultrasound‐guided choledochoduodenostomy; RBO, recurrent biliary obstruction; SEMS, self‐expandable metal stent; TRBO, time to recurrent biliary obstruction;

## DISCUSSION

We retrospectively evaluated patients who underwent EUS‐CDS using the novel SEMS with a 5.9‐Fr delivery system or conventional SEMS. In the novel SEMS group, the technical success rate without fistula dilation was 90%. No early AEs were noted. Conversely, the early AE rate in the conventional SEMS group was 19%.

In a recent systematic review, the rate of AEs following EUS‐CDS was significant (15%–22%), with bile leakage and peritonitis being the most common.^11^, ^16^ During EUS‐CDS using a conventional fully covered SEMS (conventional EUS‐CDS), the fistula dilation process and subsequent device exchange, in particular, are technically challenging and can cause bile leakage. Furthermore, the fistula dilation process is associated with the risk of guidewire displacement and technical failure.^8^, ^9^ In recent years, one‐step EUS‐CDS using lumen apposing fully covered metal stent (LAMS) had been performed to reduce AEs.^17^, ^18^, ^19^, ^20^, ^21^, ^22^ LAMS is a device specifically designed for EUS‐guided procedures. It enables target puncture, fistula dilation, and one‐step insertion of the stent delivery system. Although EUS‐CDS using LAMS is reportedly effective and safe, it remains unclear whether LAMS or conventional tubular SEMS for EUS‐CDS is superior.^23^, ^24^ On the other hand, two types of modified tubular SEMSs with thinner delivery systems had been developed, and EUS‐CDS using these devices without fistula dilation has been reported previously.^4^, ^25^, ^26^ These SEMSs had a diameter of 6 mm and 7‐Fr or 7.5‐Fr delivery systems. Their proximal end was uncovered and flared to prevent distal stent migration. The technical success rates using these SEMSs were 91%–100% in the overall cohort and 32%–100% in patients without fistula dilation. Notably, the AE rate was 0%–6%, which was considerably less than that observed in the studies using conventional EUS‐CDS.

Similarly, in this study, EUS‐CDS using the novel SEMS without fistula dilation showed no early AEs. Moreover, the novel SEMS group had a significantly lower rate of bile leakage on the CT images after the procedure and a shorter procedure time than the conventional SEMS group. EUS‐CDS without fistula dilation can be a safer procedure and can be performed in a shorter period than conventional EUS‐CDS.

We used two types of echoendoscopes: oblique‐viewing and forward‐viewing (FV) echoendoscope. Table 4 presents the outcomes and EUS findings according to the echoendoscope used in the novel SEMS group. The median length of the puncture route was significantly shorter in the FV echoendoscope group than in the oblique‐viewing echoendoscope group (7.0 mm vs. 11.5 mm, *p* = 0.02). Itonaga *et al*. reported that the length of the puncture route was significantly longer in patients who required fistula dilation than in those who did not.^26^ These findings suggest that using the FV echoendoscope is desirable to avoid the requirement of fistula dilation. However, in our study, the FV echoendoscope was used for the one patient who required fistula dilation. The length of the puncture route in the patient was 10 mm, which was the median length in the novel SEMS group. Clinically, there are possible factors in the requirement of fistula dilation, including the length of the puncture route, insertion angle of the devices, the anatomical structure of the bile ducts, and tissue hardness of the bile duct or duodenal wall. Studies with larger sample sizes are required to investigate these factors further.

**TABLE 4:** Outcomes and endoscopic ultrasound (EUS) findings according to the echoendoscope used in the novel self‐expandable metal stent (SEMS) group

	OV, *n* = 6	FV, *n* = 4	*p*
Outcomes of EUS‐CDS			
Technical success, no. (%)	6 (100)	4 (100)	1.00
Without fistula dilation	6 (100)	3 (75)	0.40
With fistula dilation	0 (0)	1 (25)	
Early adverse events, no. (%)	0 (0)	0 (0)	1.00
Procedure time in minutes, median (range)	14.5 (12–25)	22.0 (11–23)	0.07
EUS findings			
Diameter of the punctured bile duct, median, mm (range)	10.5 (7–15)	11.5 (9–19)	0.61
Length of the puncture route, median, mm (range)	11.5 (9–14)	7.0 (5–10)	0.02

Abbreviations: EUS, endoscopic ultrasonography; EUS‐CDS, endoscopic ultrasound‐guided choledochoduodenostomy; FV, forward‐viewing echoendoscope; OV, oblique‐viewing echoendoscope; SEMS, self‐expandable metal stent.

**FIGURE 5:**
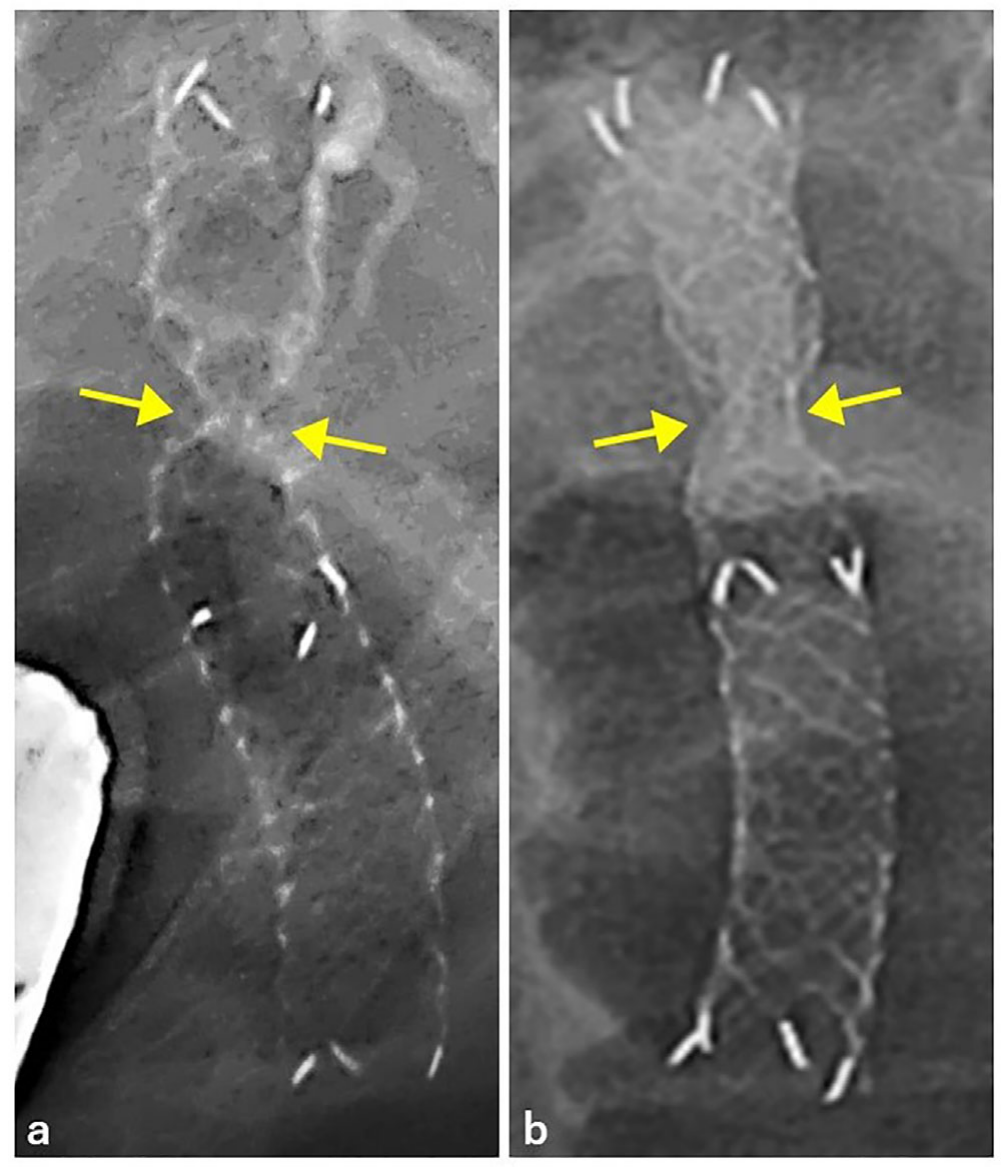
The notches immediately after deployment of the novel self‐expandable metal stent (SEMS) without fistula dilation and conventional braided fully‐covered SEMS with fistula dilation. The notch (arrows) in the novel SEMS. (a) is deeper and steeper than that in the conventional SEMS (b)

Distal migration of the novel SEMS was observed in two patients, both of whom were late‐onset cases. Theoretically, a non‐dilated fistula is considered narrower than a dilated one. In addition, the radial force of the novel SEMS is 40% lower than that of the conventional HANAROSTENT. Therefore, after deploying the novel SEMS, a deep notch was shaped at the fistula, and the risk of early migration was mitigated (Figure 5). In this study, there was no migration of the novel SEMS when pushing the distal end of the SEMS with the scope and facing it toward the anal side immediately after deployment.

The limitations of this study include its retrospective design with a limited number of patients and follow‐up periods. Two experts and 12 trainees were involved in this study, and their respective technical differences and echoendoscope choice might have introduced biases in the outcomes. In addition, the period of the procedure was different between the conventional and novel SEMS groups. Therefore, the learning curves of the echoendoscopists or their team, and the transition in the development of devices other than the SEMSs, might have affected the outcomes of the procedures. Hence, we are currently conducting a phase II prospective trial to evaluate the clinical efficacy and safety of the EUS‐CDS procedure performed in the novel SEMS group.

In conclusion, EUS‐CDS without fistula dilation using the novel SEMS with a 5.9‐Fr delivery system is technically feasible and capable of achieving straightforward, quick, and safe procedures while effectively preventing bile leakage. Although further clinical studies are needed to validate our findings, EUS‐CDS without fistula dilation has the potential to be more widely performed and could lead to safer primary drainage for many patients.

## CONFLICT OF INTEREST

The authors declare that they have no conflict of interest.

## FUNDING INFORMATION

National Cancer Center Research and Development Fund (31‐A‐13).
